# Concurrent and predictive validity of physical activity measurement items commonly used in clinical settings– data from SCAPIS pilot study

**DOI:** 10.1186/s12889-015-2316-y

**Published:** 2015-09-28

**Authors:** Örjan Ekblom, Elin Ekblom-Bak, Kate A Bolam, Björn Ekblom, Caroline Schmidt, Stefan Söderberg, Göran Bergström, Mats Börjesson

**Affiliations:** Åstrand Laboratory of Work Physiology, The Swedish School of Sport and Health Sciences, Stockholm, Sweden; School of Human Movement and Nutrition Sciences, The University of Queensland, Brisbane, QLD Australia; Department of Molecular and Clinical Medicine, University of Gothenburg, Gothenburg, Sweden; Sahlgrenska Centre for Cardiovascular and Metabolic Research, Wallenberg Laboratory, Sahlgrenska University Hospital, Gothenburg, Sweden; Department of Public Health and Clinical Medicine, Medicine and Heart Centre, Umeå University, Umeå, Sweden; Karolinska University Hospital, Stockholm, Sweden

**Keywords:** Sedentary, Prediction, Metabolic syndrome, Questionnaire, Accelerometer

## Abstract

**Background:**

As the understanding of how different aspects of the physical activity (PA) pattern relate to health and disease, proper assessment is increasingly important. In clinical care, self-reports are the most commonly used assessment technique. However, systematic comparisons between questions regarding concurrent or criterion validity are rare, as are measures of predictive validity. The aim of the study was to examine the concurrent (using accelerometry as reference) and predictive validity (for metabolic syndrome) of five PA questions.

**Methods:**

A sample of 948 middle-aged Swedish men and women reported their PA patterns via five different questions and wore an accelerometer (Actigraph GT3X) for a minimum of 4 days. Concurrent validity was assessed as correlations and ROC-analyses. Predictive validity was assessed using logistic regression, controlling for potential confounders.

**Results:**

Concurrent validity was low-to-moderate (*r* <0.35 and ROC AUC <0.7) with large misclassifications regarding time spent sitting/sedentary and in moderate-to vigorous PA. The predictive validity of the questions was good, and one question (PHAS) showed an 80 % decreased odds-ratio of having metabolic syndrome, after taking potential confounders into consideration.

**Discussion:**

In this mixed sample of adults, both concurrent and predictive validity vaired between items and between measures of the physical activity pattern. The PHAS and WALK items are proposed for assessment of adherence to PA recommendations.

**Conclusion:**

Assessing PA patterns using self-report measures results in methodological problems when trying to predict individual risk for the metabolic syndrome, as the concurrent validity generally was low. However, several of the investigated questions may be useful for assessing risk at a group level, showing better predictive validity.

## Background

In the late 1950s physical activity (PA) was somewhat simplistically dichotomized as “high” or “low”. The description of physical activity patterns has since been developed and a description of an individual’s “daily activity pattern” now commonly includes the duration and frequency, as well as intensity of the activity (from sedentary behaviour to light to vigorous intensity). The understanding of the relationships between bodily movement and health outcomes has improved greatly, revealing the increasing number of health conditions found to be closely related to PA.

Metabolic syndrome (MetS) is a cluster of conditions in which several aspects of energy storage and utilization are altered, leading to an increased risk for cardiovascular and metabolic diseases. Previous studies have indicated that PA is associated with risk for MetS [1, 2]. Therefore, assessing the predictive validity (i.e. the ability to predict an outcome, rather than to assess a behaviour) of commonly used PA questions to examine individual and group risk for health risk factors is of great clinical importance.

In clinical practice, the use of objective measures, are restricted for many reasons, including logistical complexities, the high costs associated and a lack of knowledge on interpretation of data gained from these measures. Consequently, self-report PA instruments originally intended for studies in large populations, are often used to screen and monitor activity patterns of patients in clinical settings.

While the concurrent validity of self-reported PA has been shown to be limited [3, 4], the predictive validity may well be comparable to objective measures. Indeed, the present international PA recommendations are largely based on self-reported data [5, 6]. Both the concurrent and predictive validity of self-reported PA measures are of clinical interest when examining an individual’s PA pattern or possible changes herein and when predicting overall risk of disease.

Self-report methods of PA measurement typically involve asking the respondent to either rank their activity using a number of predefined categories or to report time spent in a certain activity during a week or other time unit. A number of self-report forms have been assessed for validity. The four levels of the single-item Saltin-Grimby Physical Activity Level Scale (SGPALS) have been shown to be associated to cardiovascular risk factors [7] as well as predicting mental health [8] and cerebrovascular morbidity and mortality [9]. Widely used in Sweden, the single question on PA is used by the Public Health Agency of Sweden to estimate general PA level within the population, however this question has not yet been validated.

In summary, self-report questionnaires have seldomly been simultaneously examined regarding both concurrent and predictive validity. Thus, it is still not clear if any of these types of measures are related to objectively measured PA or have superior predictive validity for health outcomes. Therefore, the first aim of the present study was to assess the concurrent validity of self-report questions of PA, using accelerometry as an objective reference measure. The second aim was to assess the predictive validity of the self-report questions for MetS.

## Methods

Objective (accelerometer) and subjective (self-report questions) measures of PA, anthropometry, haemostatic and metabolic data were derived from the Swedish CArdioPulmonary bioImage Study (SCAPIS) pilot study, conducted at the Sahlgrenska University Hospital in Göteborg, Sweden. The participants completed an extensive questionnaire regarding lifestyle and living conditions, as well as performed a submaximal cycle test to determine cardiorespiratory fitness. Participants underwent an examination, including blood pressure, waist circumference, blood lipids (triglycerides and high-density lipoprotein) and fasting glucose. Further, participants wore an accelerometer during seven days to objectively assess activity patterns. The study, was approved by the Umeå ethical board (Dnr 2010-228-31M). All participants provided written informed consent.

## Study population

A randomly selected population sample of 2243 adults aged 50–65 years from low and high socioeconomic status areas were drawn from the Swedish national census register. Out of the original 2243, 1111 agreed to participate in the SCAPIS study.

## Measurement of sedentary behaviour and physical activity

### Self-report questions

Five different instruments were analysed in this study. Two of these focus on habitual physical activity (A) the “PHAS” question created and used nation-wide, by the Public Health Agency of Sweden [10] and (B) a Swedish version of the SGPALS [11]. The PHAS and the SGPALS required respondents to answer with fixed response alternatives. Additionally, two items assessed moderate- to vigorous PA, (MVPA), (C) and walking (D), and responses were expressed as free values in minutes per week performing MVPA and walking. The fifth instrument assessed time spent sitting (sedentary) (E), where the respondent was asked to report in free values minutes per day and days per week of sitting.

A. The PHAS instrument [10] asked “*How much have you moved about and exerted yourself physically in your leisure time in the last 12 months? If your activity varies between eg summer and winter, try to take an average”*. Response alternatives were as follows:
  *1 = Sedentary leisure time. You spend most of your time reading, television, cinema or other sedentary activities in leisure time. You walk, cycle or move about in other ways less than 2 h a week;*
  *2 = Moderate but regular exercise during leisure time. You exercise regularly 1–2 times per week for at least 30 min at a time, e.g. running, swimming, tennis, badminton or other activity that makes you sweat;*
  *3 = Moderate exercise during leisure time. You walk, ride a bicycle or move about in other ways, for at least 2 h a week without sweating. This includes e.g. walking or cycling to and from work, other walks, heavier household work, normal gardening, fishing, tennis or bowling;*
  *4 = Regular exercise and training. You participate in for example running, swimming, tennis, badminton, exercise gymnastics or similar at least 3 times per week. Each session lasts at least 30 min at a time.*
B. The SGPALS instrument [11] asked “*How much do you move and exert yourself physically during leisure time? If your activity varies greatly between, for example summer and winter, try to estimate an average. The question concerns the last 5 years*”. Response alternatives were as follows:1*.****Physically inactive****(I): Being almost completely inactive, reading, watching television, watching movies, using computers or doing other sedentary activities, during leisure-time.*
  *2.****Some light physical activity****(LPA): Being physically active for at least 4 h/week as riding a bicycle or walking to work, walking with the family, gardening, fishing, table tennis, bowling etc.*
  *3.****Regular physical activity and training****(moderate PA, MPA): Spending time on heavy gardening, running, swimming, playing tennis, badminton, calisthenics and similar activities, for at least 2 to 3 h/week.*
  *4.****Regular hard physical training****for competition sports (vigorous PA, VPA): Spending time in running, orienteering, skiing, swimming, soccer, European handball etc. several times per week.*

The questions on the quantification of time spent in MVPA (C), walking (D), and sitting (sedentary behaviour) (E), respectively, focused on the last 7 days. More in detail, these questions read:

- *C1: During the past 7 days, have you performed work that is moderately strenuous as cycling, swimming, moderately exerting construction and gardening or other activities of moderate intensity? Do not include walking*. (moderate physical activity)
- *C2: During the past 7 days, have you performed activities that are very strenuous, such as heavy lifting, heavy construction and gardening, aerobics, running or biking in higher pace?* (vigorous activity)
- *D. During the past 7 days, you have spent time walking, at least 10 min at a stretch?* (walking)
- *E. During the past 7 days, you have spent time sitting during this period?* (sedentary)

If responding “yes” to any of the questions C-E, participants thereafter reported the number of days per week and the number of hours per day, performing these activities. Hereafter, minutes of each activity per week were calculated as a product of these two arguments, multiplied by 60. Total time in MVPA was calculated from the total time (min per week) in moderate and vigorous activities.

### Accelerometry

ActiGraph accelerometers (model GT3X and GT3X+, ActiGraph LCC, Pensacola, FL, USA) were used to objectively measure activity patterns. The accelerometer was handed to the participant during the second day of the visits to the test centre. Participants were instructed to wear the accelerometer on an elastic belt over the right hip during all waking hours for at least seven consecutive days, except during water-based activities. After the measurement period, the accelerometer was returned by prepaid mail. The accelerometer was initialized and data was downloaded using the ActiLife v.6.10.1 software. Raw data sampling frequency was set to 30 Hz, and extracted as 60-s triaxial epochs with low frequency extension filter for the analyses.

### Accelerometer data processing

Of the 1111 participants who agreed to participate in the SCAPIS study, a total of 1067 participants agreed to wear an accelerometer. Minimum requirement for inclusion in the analysis was 600 min of valid daily monitor wear, on at least 4 days. Wear time was defined by subtracting non-wear time from 24 h. Non-wear time was defined as at least 60 consecutive minutes with no movement, 0 counts per minute (cpm), with allowance for maximum 2 min of movement with intensities up to 200 cpm. The majority of the included participants had valid data for at least 7 days (67 %), 19 % for 6 days, 9 % for 5 days, and 5 % for 4 days. A total of 948 participants had valid data.

The daily activity pattern is described using the following components 1) percentage wear time spent in three intensity-specific categories; sedentary (SED), light PA (LIPA) and MVPA. 2) total volume of PA expressed as mean cpm over the study period (TPA), 3) time spent in prolonged periods of SED (bouts of ≥20 consecutive minutes below SED threshold, with no allowance for interruption above threshold) (SED bouts), 4) fulfilment of Swedish national PA recommendations (see below).

#### Accelerometer analysis

Accelerometer data was analysed as triaxial data, using the vector magnitude as accelerometer output. Using standard definitions, time was classified as SED when spent in intensities between 0 and 199 cpm [12], LIPA was regarded as cpm between 200 and 2689, and MVPA as ≥2690 cpm, [13]. Mean vector magnitude cpm was used as an expression of TPA and was calculated as total vector magnitude cpm divided by minutes of wear time.

The time in sedentary activities as measured by accelerometry was used to assess concurrent validity for time spent sitting. These two entities are not equivalent but express the same type of activities to a large degree. Onwards, the term sedentary will be collectively used to describe both the self-reported and the objectively assessed behaviour.

#### National recommendations

Current Swedish national PA guidelines [6] recommend at least 150 min of MVPA per week, preferably spread out over most days of the weeks. The activity can be divided in bouts of 10 min or more. We chose to evaluate the relationship between reaching the guidelines and MetS prevalence, using a strict interpretation of the recommendations (30 min per day on at least 5 of 7 days of the week, of which all are from prolonged bouts of 10 min or more) and also a less strict interpretation of the recommendations, excluding the requirement of regularity and accumulation in prolonged bouts (accumulating a total of 150 MVPA minutes per week).

## Fitness testing

All participants were invited to perform a submaximal cardiorespiratory fitness test [14]. A total of 130 participants (14.1 %) did not participate, mainly due to pain (knee, lower back and hip), body mass above the maximal user weight for the cycle ergometer (125 kg), perceived inability to perform a test, on-going illnesses or due to malfunction of heart rate monitors or the cycle ergometer. Cardiorespiratory fitness (CF) was estimated based on the difference in heart rate response between a lower and a higher submaximal work rate, and expressed as mL·min^−1^·kg^−1^.

## Metabolic syndrome

Participants were classified as either having or not having MetS, according to the National Cholesterol Education Program (NCEP), 2001 definition [15]. The NCEP Adult Treatment Panel III (ATPIII panel), defined MetS as the presence of three or more of the following: fasting plasma glucose ≥6.1 mmol·l^−1^, serum triglycerides ≥1.69 mmol·l^−1^, serum HDL-cholesterol ≤1.04 mmol·l^−1^, in men and ≤1.29 mmol·l^−1^ in women, blood pressure ≥130/85 mmHg, or waist circumference ≥102 cm in men and ≥88 cm in women. For details see [16].

## Other measurements

Measurements of weight, height and waist circumference were performed during the first visit to the test centre. Through self-administrated questionnaire responses, education level was dichotomized into gaining university degree or not, smoking habits dichotomized into regular vs. ex-smoker/never-smoker, and perceived psychosocial stress (reporting tension, anxiousness, nervousness or sleep disturbances more or less constantly over the last year or longer) divided into four levels. An extensive food frequency questionnaire was used to assess food habits, and answers were used to calculate daily caloric intake (EI).

## Statistics

All statistical analyses were performed using SPSS (Statistical Package for the Social Sciences for Windows, 14.0, 2006, SPSS Inc., Chicago IL).

The descriptive data is presented as proportions or median and 25th–75th percentile (Q1–Q3). Differences between genders were tested with Chi-square and Mann–Whitney U tests.

Concurrent validity was assessed using correlation analysis or Spearman’s rho if appropriate, to examine for potential relationships between self-reported (categories and minutes per week, respectively) and accelerometer derived data. Further, as data was skewed, misclassification was assessed as median (5th – 95th percentile) difference between self-reported and accelerometer derived sedentary time and MVPA respectively. Finally, receiver characteristics curve (ROC) analysis was performed to assess the ability of the five different instruments, to correctly classify participants meeting and not meeting the two interpretations of the Swedish National PA recommendations. ROC data is presented as area under the curve (AUC) with the 95 % confidence intervals as well as sensitivity and specificity for the tested self-reports.

For predictive validity analyses, self-reported data were arbitrarily divided into four strata. For PHAS and SGPALS, these strata were the four response alternatives and for walking (WALK), MVPA and sedentary time, sex-specific quartiles were used. Frequencies in the stratas were 140, 399, 249 and 148 for PHAS and 99, 321, 380 and 10 for SGPALS, thus showing a slightly higher attrition rate for SGPALS. Odds ratios (ORs) with 95 % confidence interval (95 % CI) for having MetS were calculated using binominal logistic regression, controlling for a) age and gender and b) age, gender, educational level, smoking status, psychological stress and EI. For each self-report, reference value was set as the lowest quartile, i.e. lowest amount of MVPA or lowest amount of sedentary time. When the 95 % CI did not include the reference value of 1, ORs were considered as significantly elevated or lowered.

## Results

Table 1 shows participant characteristics. Metabolic syndrome was found in 20.4 and 23.5 % of women and men, respectively. A higher proportion of women than men reported high stress. Men reported a higher energy intake, compared to women. Reported sedentary time was lower than accelerometer derived. Conversely, the reported time spent in MVPA was higher than the values obtained by accelerometry (i.e. misclassification).

**Table 1:** Participant characteristics with data presented as median (25–75 percentile) or as percent

Variable	Men	Women
	*n* = 462	*n* = 486
Median age (yrs)	57.7 (53.8–62.0)	57.5 (53.7–61.4)
Height (cm)	178 (173–183)	165 (160–169) ^b^
Body mass (kg)	86.6 (79.5–95.0)	70.4 (63.7–80.0) ^b^
Waist circumference	99 (94–105)	89 (81–98) ^b^
Fitness (mL × min^−1^ × kg^−1^)	38.9 (35.8–43.3)	29.2 (24.1–33.8) ^b^
MetS	23.5 %	20.4 %
University education	34.6 %	41.5 % ^b^
Regular smokers	12.9 %	11.2 %
High stress^a^	15.3 %	27.1 % ^b^
Median reported energy intake (kcal per day)	1910 (1456–2467)	1637 (1275–2146) ^b^
Median time spent walking	25.7 (8.6–60)	34.3 (17.1–60) ^b^
Median time in LIPA	329 (280–385)	278 (323.431) ^b^
Median sedentary time (min per day)		
Self-report	300 (180–420)	240 (180–420) ^b^
Accelerometer	528 (470–589)	502 (438–560)
Median MVPA (min per day)		
Self-report	180 (0–480)	120 (0–360) ^b^
Accelerometer	35.4 (21.4–49.5)	30.8 (19.3–45.6)

^**a**^ Reporting tension, anxiousness, nervousness or sleep disturbances more or less constantly over the last year or longer

^b^ significant difference between genders (Chi-square or Mann–Whitney U tests)

Self-reported MVPA is based on questionnaire C and sedentary time is based on questionnaire E

*MetS* Metabolic syndrome, *LIPA* Light physical activity, *MVPA* Moderate-to-vigorous physical activity

## Concurrent validity

The agreement between self-reports and objectively assessed PA was low to moderate (Table 2). The strongest correlations were found for the PHAS question vs. MVPA and for the self-reported time spent sedentary and SED or LIPA (negative relation). The answers from the two questions with fixed categories showed low correlations to SED and LIPA. The answers from the open questions predicted low intensity activities better, as shown in Table 2.

**Table 2:** Concurrent validity, expressed as correlation (Spearman’s rho) for accelerometer derived and self-reported estimates of PA and sedentary behaviour

	Accelerometer PA					
Self-reported PA	SED	LIPA	MVPA	TPA	SED bouts	CF
Total leisure time PA						
PHAS	**−0.12**	0.04	**0.31**	**0.26**	**−0.12**	**0.22**
SGPALS	**−0.07**	0.01	**0.23**	**0.21**	−0.07	**0.17**
WALK	**−0.20**	**0.14**	**0.24**	**0.26**	**−0.07**	**0.02**
MVPA	**−0.12**	**0.10**	**0.14**	**0.15**	**−0.04**	**0.16**
Sedentary	**0.30**	**−0.32**	−0.03	**−0.19**	**−0.20**	0.07

Values in bold denotes *p* <0.05

*PA* Physical activity, *PHAS* PA-question by the Public Health Agency of Sweden, *SGPALS* Saltin-Grimby Physical Activity Level Scale, *WALK* Min per day of walking, *MVPA* Min per day of moderate-to-vigorous PA, *SED* % time spent sedentary (via accelerometer), *LIPA* % time spent in light intensity PA (via accelerometer), *MVPA* % time spent in moderate to vigorous PA (via accelerometer), *TPA* Total volume of PA expressed as mean cpm over the study period (via accelerometer), *SED bouts* Total time spent sedentary in prolonged bouts (>20 min), CF Cardiorespiratory fitness

**Fig 1:**
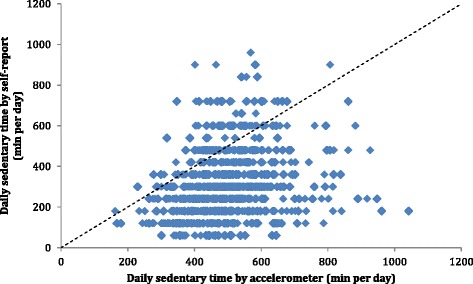
Scatterplot for objectively measured (accelerometry) and self-reported time spent sedentary by question E. The line of identity is plotted in the figure. Spearman’s rho was 0.30 (*p* <0.001)

**Fig 2:**
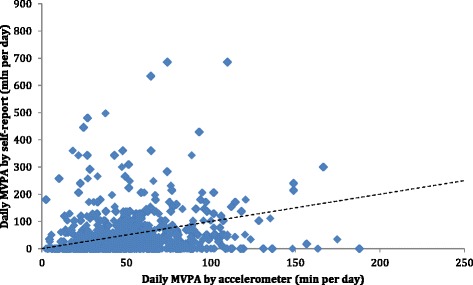
Scatterplot for objectively measured (accelerometry) and self-reported time spent in moderate-to-vigorous physical activity (MVPA, minutes per week, from question C). The line of identity is plotted in the figure. Spearman’s rho was 0.14 (*p* <0.001)

As indicated visually in Figs. 1 and 2, weak relationships were present, between reported sedentary time and objectively measured time spent being sedentary as well as time spent in MVPA. The median misclassification of time spent sedentary, calculated as self-report minus accelerometry was −185 (5th–95th percentile: −467 to 119) minutes per day. From the self-reports, 10.5 % of the participants reported no sedentary time, while the lowest accelerometer derived average time spent in sedentary activity was 162 min per day. The median misclassification of time spent in MVPA, calculated as self-report minus accelerometry, was −21.1 (5th–95th percentile: −81.1 to 111.1) minutes per day.

Concurrent validity was also assessed using fulfilment of the two interpretations of the Swedish National PA recommendations (Table 3). Regarding the first interpretation, the PHAS question showed the highest AUC (0.70, 95 % CI: 0.66 to 0.74). The highest AUC for the second interpretation was found for the WALK question (0.70: 95 % CI: 0.64 to 0.76).

**Table 3:** Sensitivity, specificity and area under the curve (95 % CI) for each self-report for identifying participants meeting PA recommendations (assessed by accelerometry)

	AUC	95 % CI	Sensitivity	Specificity
A. MVPA 150 min per week				
PHAS	0.70	(0.66 − 0.74)	92 %	27 %
SGPALS	0.64	(0.59 – 0.68)	55 %	70 %
MVPA	0.57	(0.54 – 0.63)	62 %	56 %
WALK	0.61	(0.55 – 0.66)	70 %	48 %
Sedentary time	0.51	(0.47 – 0.56)	54 %	48 %
B. MVPA 30 min per day in >10 min bouts on at least 5 of 7 days of the week				
PHAS	0.59	(0.52 – 0.66)	56 %	56 %
SGPALS	0.65	(0.58 – 0.72)	75 %	54 %
MVPA	0.52	(0.44 – 0.60)	54 %	46 %
WALK	0.70	(0.64 – 0.76)	84 %	48 %
Sedentary time	0.59	(0.52 – 0.66)	64 %	47 %

*PA* Physical activity, *PHAS* PA-question by the Public Health Agency of Sweden; *SGPALS* Saltin-Grimby Physical Activity Level Scale, *WALK* Min per day of walking, *MVPA* Min per day of moderate-to-vigorous PA

## Predictive validity

In addition to the agreement with accelerometer derived PA, the ability of the five questions to predict the presence of MetS was studied (Table 4). Odds ratios (ORs) for having MetS were calculated for four strata of self-reported PA and sedentary behaviour. Odds ratios were adjusted for age and gender in the first model and for age, gender, education level, EI, smoking and psychosocial stress, in the second. Compared to the lowest level of PA (S1, reference group), the fourth strata (S4, most active as measured by the PHAS-question) had a near 80 % risk reduction in the second model (OR = 0.23). Similar values were found for SGPALS, but with a wider confidence interval and thus non-significant, possibly due to fewer respondents in S4 (most active). Relationship between MVPA and MetS was weaker, but still significant. WALK did not predict MetsS. respectively for S4). No significant relationship was found between self-reported sedentary time (where S1 represented the lowest time spent sedentary) and presence of MetS.

**Table 4:** Odds ratios (95 % CI) for MetS in four strata of self-reported PA or sedentary behaviour. First quartile or strata acted as reference, with participants reporting the lowest levels

		S2	S3	S4
PHAS	Age-gender	**0.45 (0.30**–**0.69)**	**0.33 (0.20**–**0.53)**	**0.17 (0.09**–**0.33)**
	Lifestyle	**0.54 (0.34**–**0.86)**	**0.41 (0.24**–**0.70)**	**0.23 (0.11**–**0.46)**
SGPALS	Age-gender	**0.57 (0.34**–**0.94)**	**0.37 (0.22**–**0.62)**	0.22 (0.03–1.79)
	Lifestyle	0.64 (0.37–1.11)	**0.41 (0.23**–**0.73**)	0.34 (0.04–2.92)
MVPA (min.d^−1^)	Age-gender	0.42(0.14–1.27)	**0.42 (0.28**–**0.63)**	**0.50 (0.33**–**0.75)**
	Lifestyle	0.52 (0.17–1.59)	**0.49 (0.32**–**0.75)**	**0.50 (0.33**–**0.78)**
WALK (min.d^−1^)	Age-gender	1.20 (0.76–1.89)	0.67 (0.44–1.01)	1.00 (0.62–1.59)
	Lifestyle	1.26 (0.74–1.99)	0.66 (0.42–1.04)	1.04 (0.63–1.74)
Sedentary time (min.d^−1^)	Age-gender	0.95 (0.61–1.50)	0.89 (0.59–1.34)	0.69 (0.42–1.12)
	Lifestyle	1.32 (0.80–2.18)	1.18 (0.75–1.88)	0.97 (0.56–1.66)

ORs in bold denotes values different from reference group

- Age-gender: Adjusted for age (yrs) and gender

- Lifestyle: adjusted for age (yrs), gender, education level (university level vs. lower), energy intake (kcal/d), smoking (regular vs. ex-smoker/never-smoker) and psycho-social stress

- Quartile limits for MVPA/WALK/sedentary were 0/8.6/42.9 min per day, 17.1/34.3/60 min/day and 60/180/360 min per day, respectively for women. For men, corresponding values were 0/17.1/60; 8.6/25.7/60, and 60/180/360 min/dag, respectively

*PA* Physical activity, *PHAS* PA-question by the Public Health Agency of Sweden, *SGPALS* Saltin-Grimby Physical Activity Level Scale, *WALK* Min per day of walking, *MVPA* Min per day of moderate-to-vigorous PA

*S2, S3 and S4* Second, third and fourth strata or quartile, respectively

## Discussion

The main findings of the present study are that the PA questions frequently used in clinical settings, all showed weak to moderate relationships with the objective PA assessment method, accelerometry. Thus, the concurrent validity of the questionnaires was found to be low. In spite of this, the predictive validity of the questions with fixed answering alternatives (PHAS and SGPALS) was moderate to high for predicting MetS, although the highest quartile for SGPALS did not differ from the lowest (possibly due to a low number of values). For example, the most active group on the PHAS-scale had a near 80 % decreased odds-ratio of having MetS (OR = 0.23), compared to the least active group. These findings have potential important clinical implications, as PA assessment using self-reports, has been advocated for use in health care, for risk assessment as well as for individual PA prescription/counselling [17, 18]. However, when analysing the data from these self-report measures it is vital to consider that the large median bias between reported and objectively assessed PA reduces the usefulness of questionnaires, on an individual level.

Regarding concurrent validity, the strongest relationships were found between PHAS responses and objectively assessed time spent in MVPA or TPA, and between self-reported sedentary time and objectively assessed SED and LIPA. The latter is somewhat surprising, as earlier reports typically [19] find low concurrent validity for self-reported sedentary time. However, the large absolute misclassification (median bias 185 min per day, 5th to 95th perc: −467 to 119 min per day) makes the usefulness in an individual case problematic. Large misclassification was also found for MVPA, albeit to a lesser absolute extent. When expressed as a proportion of the objectively assessed time in the respective intensity, misclassification was higher, almost doubled, for MVPA compared to SED.

The low to moderate correlations between the questions (PHAS, SGPALS and MVPA) and objectively assessed MVPA, are in line with earlier studies [3, 4]. None of the studied questions met the criterions for level 1 or level 2 evidence (*r* >0.5 using accelerometer as reference method) according to van Poppel and colleagues [20] or standard for measurement properties of PA questionnaires as stated in the Quality Assessment of Physical Activity Questionnaire (*r* >0.5 for TPA, MPA or VPA, or *r* >0.7 for WALK) [21]. This implies that their use for judging individual PA level or pattern is, *per se*, limited. The MVPA and WALK questions are similar, although not identical to the International Physical Activity Questionnaire (IPAQ) [22]. Our results show slightly higher correlations to objective measures than has previously been reported for the IPAQ in Swedish adults [23]. While having high concurrent validity is one important aspect of quality for an assessment instrument, perhaps the most important characteristic for PA questionnaires used in clinical is their validity to predict a patient’s risk for unwanted health outcomes.

Although the median misclassification indicate a general overestimation of time spent in MVPA, a rather large proportion of the participants underestimated time in MVPA. An explanation for this may be the rather low limit of what constitutes “moderate intensity”, which starts at 3 times the resting metabolic rate (3 METs). For example, in a normal weight woman, this equals to an oxygen consumption of approximately 0.6–0.75 l per minute or a caloric expenditure of 3–4 kcal per minute. This may be is easily accomplished during many daily activities (such as transport or household chores), rather than structured exercise. Such activities may therefore pass unnoticed and is not consciously associated with the term “moderate” when responding to a PA question, leading to en underestimation in some participants.

According to the results of the present study, using open alternatives may be a barrier for the respondent to accurately judge the actual sedentary time. While the self-reported sedentary time was not related to presence of MetS in the present analyses, Katzmarzyk and colleagues have reported [24] a strong relationship between self-reported time spent sedentary and mortality, in a Canadian cohort, using fixed response alternatives. The difference in results may stem from the difference in design or outcome. Similar results, as those presented by Katzmarzyk have earlier been shown by Matthews and colleagues [25].

Our data and others [26] indicate that self-reported time spent sedentary are greatly underestimated. Typically, adults report 5–6 h per day of sedentary time, while objective measures indicate values close to 9 or 10 h per day, which equates to a 40–50 % underestimation or misclassification. This issue must be considered when drawing conclusions on PA levels, found in various studies, in which the time spent sedentary is often assessed using similar questions to the ones used in the current study. For example, in the Eurobarometer-study [27] the sitting question from IPAQ was used. The authors reported an average time spent sitting of 309 min per day (SD 184 min/day) and similar values were found in a study [28], reporting on sitting time in 20 countries (median time spent sitting was estimated at 300 min per day). In the light of the excessive mismatch in our present data and others [26], both absolute levels and geographical variations in the Eurobarometer findings, may therefore be questioned, because of the potential for misclassification of PA when using self-report measures.

The ability of the studied questions to correctly identify participants meeting PA recommendations was low. The WALK-question was better at predicting the more strict interpretation of the current PA recommendations, while PHAS better at predicting the less strict interpretation. The sedentary question did not significantly differ from chance in predictive power regarding the less strict interpretation. This is a key limitation of self-report measures of PA, as the identification of individuals meeting or not meeting PA recommendations, is commonly used for risk assessment of certain health outcomes.

However, the predictive validity for the questions was tested as ability to predict the presence of MetS. Importantly, a clear dose–response relation was found for the PHAS-question, with falling ORs in more active strata. Despite their similarities, the PHAS and the SGPALS differed with regards to the number of responders in the most active group. While the SGPALS describe the most active group as performing “regular hard physical training”, the PHAS uses the phrasing “Regular exercise and training”, which may not be perceived as equally demanding or taxing, by the respondents. This resulted in a similar point estimate (OR), but lower precision (wider CIs) for the SGPALS. Among the open answer option questions, only MVPA showed strong correlation to MetS, with the third strata in the WALK-question showing border line significance. No significant relation was found, for reported time spent sedentary.

Our results thus imply that a fixed answer category seems more valid for identifying individuals at risk for MetS, compared to open ones. Hypothetically, this may be due to a better ability among respondents to rank themselves in a group, rather than giving a correct estimate of their time use. However, future research must compare more similarly formulated questions to be able to further make this distinction.

### Limitations

The food frequency questionnaire used returned rather low energy intake values, most probably due to an underreporting. The absolute values may therefore not be comparable to those obtained using other methods. Although, the sedentary question in this study gives the respondents a possibility to respond in hour and minutes freely, medians and percentiles in many large studies are divisible by 60 (see for example [28]), indicating that it is uncommon that respondents use the minute option and respond in full hours only. This may result in underestimation of present differences between groups.

The number of participants varied between questionnaires. Some questions may be regarded as easier to respond to and this should be taken into consideration when assessing the validity on a question or questionnaire. If including full-sample participants only (i.e. only those who provided data from all questions) the results were the same, with regard to significances and ranking between questionnaires. Therefore, the more naturalistic approach was chosen.

This cross-sectional study cannot find causality regarding physical activity and the presence of MetS. Furthermore, the use of accelerometers is to be regarded as another indirect method, not a “100 % golden standard” reference method of PA. While not really a limitation, it is important to note that, to a large degree, sitting and sedentary are similar behaviours, but may differ in some cases and thereby describe differing activities. In the present study, we used the term sedentary for both entities.

## Conclusion

The rather low concurrent validity of clinically used PA questionnaires, found in the present study is of great importance, because such questions are routinely used for PA assessment in regular health care. When comparing risk for health outcomes between different kinds of exposures, differences in concurrent validity between questionnaires may hamper comparisons. In many studies, other risk factors (e.g. blood lipids, anthropometry), are typically assessed by methods with considerably higher concurrent validity than PA questions. As methods with low concurrent validity will often underestimate the effect in comparisons to other risk factors, due to dilution, the effects of PA may be underestimated. The effect of the different sub-components of the PA pattern, assessed with inferior criterion validity, may also be underestimated. It is therefore concerning, that the studied questions poorly predicted fulfilment of current PA recommendations.

It is important to remember that international treatment recommendations are based on self-reported PA assessments. Furthermore, the predictive validity of outcomes, such as morbidity or mortality is the most important for the patient, not the exact amount of PA undertaken. In this study, the questions using fixed alternatives seem to have the highest predictive validity, while having similar concurrent validity to the other questions, although this has to be further studied. Thus, the stronger predictive validity for the questionnaires, using fixed answer alternatives (PHAS and SGPALS), indicate that these questions are clinically most useful, and this should be considered by health professionals when choosing questionnaires to assess PA.

## Abbreviations

PA: Physical activity
ROC: Receiver characteristics curve
AUC: Area under the curve
PHAS: Public Health Agency of Sweden
MetS: Metabolic Syndrome, in the present paper according to National Cholesterol Education Program, Adult Treatment Panel III
SGPALS: Saltin-Grimby physical activity level scale
SCAPIS: Swedish CArdioPulmonary bioImage study
MVPA: Moderate- to vigorous PA
SED: Sedentary
LIPA: Light intensity PA
WALK: Time spent walking
Cpm: Counts per minute, i.e. accelerometer data expressing activity intensity
TPA: Total physical activity
CF: Cardiorespiratory fitness
HDL: High density lipoprotein
EI: Daily caloric (energy) intake
OR: Odds ratio
CI: Confidence interval
